# Clinical trial protocol for TARDOX: a phase I study to investigate the feasibility of targeted release of lyso-thermosensitive liposomal doxorubicin (ThermoDox®) using focused ultrasound in patients with liver tumours

**DOI:** 10.1186/s40349-017-0104-0

**Published:** 2017-11-02

**Authors:** Paul C. Lyon, Lucy F. Griffiths, Jenni Lee, Daniel Chung, Robert Carlisle, Feng Wu, Mark R. Middleton, Fergus V. Gleeson, Constantin C. Coussios

**Affiliations:** 1 Oxford Institute of Biomedical Engineering, University of Oxford, Oxford, UK; 2 Nuffield Department of Surgical Sciences, John Radcliffe Hospital, Headington, Oxford, UK; 3 Department of Radiology, Oxford University Hospitals NHS Foundation Trust, Oxford, UK; 4 Oncology Clinical Trials Office, Department of Oncology, University of Oxford, Oxford, UK; 5 Department of Oncology, Oxford University Hospitals NHS Foundation Trust, Oxford, UK

**Keywords:** FUS, HIFU, Therapeutic ultrasound, Focused ultrasound, ThermoDox®, Lyso-thermosensitive liposomal doxorubicin (LTLD), Targeted drug delivery, Triggered release, Liver tumour(s)

## Abstract

**Background:**

TARDOX is a Phase I single center study of ultrasound triggered targeted drug delivery in adult oncology patients with incurable liver tumours. This proof of concept study is designed to demonstrate the safety and feasibility of targeted drug release and enhanced delivery of doxorubicin from thermally sensitive liposomes (ThermoDox®) triggered by mild hyperthermia induced by focused ultrasound in liver tumours. A key feature of the study is the direct quantification of the doxorubicin concentration before and after ultrasound exposure from tumour biopsies, using high performance liquid chromatography (HPLC).

**Methods/Design:**

The study is conducted in two parts: Part 1 includes minimally-invasive thermometry via a thermistor or thermocouple implanted through the biopsy co-axial needle core, to confirm ultrasound-mediated hyperthermia, whilst Part 2 is carried out without invasive thermometry, to more closely mimic the ultimately intended clinical implementation of the technique. Whilst under a general anaesthetic, adult patients with incurable confirmed hepatic primary or secondary (metastatic) tumours receive a single cycle of ThermoDox®, immediately followed by ultrasound-mediated hyperthermia in a single target liver tumour. For each patient in Part 1, the HPLC-derived total doxorubicin concentration in the ultrasound-treated tumour is directly compared to the concentration before ultrasound exposure in that same tumour. For each patient in Part 2, as the tumour biopsy taken before ultrasound exposure is not available, the mean of those Part 1 tumour concentrations is used as the comparator. Success of the study requires at least a two-fold increase in the total intratumoural doxorubicin concentration, or final concentrations over 10 μg/g, in at least 50% of all patients receiving the drug, where tissue samples are evaluable by HPLC. Secondary outcome measures evaluate safety and feasibility of the intervention. Radiological response in the target tumour and control liver tumours are analysed as a tertiary outcome measure, in addition to plasma pharmacokinetics, fluorescence microscopy and immunohistochemistry of the biopsy samples.

**Discussion:**

If this early phase study can demonstrate that ultrasound-mediated hyperthermia can effectively enhance the delivery and penetration of chemotherapy agents intratumorally, it could enable application of the technique to enhance therapeutic outcomes across a broad range of drug classes to treat solid tumours.

**Trial registration:**

ClinicalTrials.gov Identifier: NCT02181075, Edura-CT Identifier: 2014-000514-61.

Ethics Number: 14/NE/0124.

## Background

A major challenge of systemic chemotherapy is the delivery of a therapeutic dose to the target tumour without exceeding the maximum tolerated dose in other tissues. Device-targeted drug delivery has seen decades of pre-clinical development but could now be at the dawn of clinical adoption, as a generic tool for overcoming the challenges of delivering existing and emerging therapeutics to solid tumours [1–3]. A significant body of pre-clinical research has demonstrated that hyperthermia-triggered release of a therapeutic agent encapsulated in thermosensitive carriers can greatly enhance the intratumoural concentration of available drug, tumour penetration and ultimate therapeutic efficacy for a given systemic dose [4–9], but these effects have not yet been demonstrated clinically.

Small animal tumour models have demonstrated that MR guided HIFU systems can non-invasively generate tissue hyperthermia, releasing circulating low thermosensitive liposomes (LTSLs), as confirmed by co-encapsulation with paramagnetic MRI contrast agents (“dose painting”) [10–14]. ThermoDox® (Celsion Corporation, USA) is a specially formulated and long-circulating Lyso-Thermosensitive Liposomal Doxorubicin (LTSD) approved for investigational use, and contains a broad-spectrum cytotoxic agent (doxorubicin). To date, ThermoDox® has predominantly been used clinically in conjunction with Radiofrequency Ablation (RFA), whereby RFA is used to ablate the core of the tumour and doxorubicin is intended to improve therapy of the tumour margins. Safety of ThermoDox® was first demonstrated as part of the HEAT trial, a 701-patient pivotal Phase III study. Although the study did not meet its primary endpoint of a 33% improvement in progression-free survival (with an 80% power and *p* value of 0.05), post-hoc subgroup analysis demonstrated a 53% risk improvement in overall survival in the subset of patients having received RFA treatment for 45 min or more with ThermoDox®. The ongoing OPTIMA study is a lead Randomized, Double Blind, Dummy-Controlled Phase III clinical study of ThermoDox® used in combination with standardized RFA for 45 min or more for solitary Hepatocellular Carcinoma (HCC). The OPTIMA study was initiated in June 2014 and is estimated to enrol about 550 patients. The primary endpoint is overall survival with progression free survival as a secondary endpoint. ThermoDox® is also currently under investigation in recurrent chest wall cancer, where a superficial hyperthermia device is placed in contact with the skin (DIGNITY study) and in paediatric solid tumours in combination with HIFU.

Unlike RFA, focused ultrasound (FUS) is a non-invasive clinical treatment modality. FUS has been shown to be safe [15, 16], highly targeted and is repeatable due to its non-ionising nature. When used at high intensities, it is capable of direct and instantaneous solid tumour ablation at depth in the body. FUS has demonstrated excellent clinical safety profiles in a range of abdominopelvic target organs, including the liver, kidneys, pancreas and uterus and is under investigation for sarcoma and intra-cranial applications. When used at lower intensities, it is capable of generating highly targeted mild hyperthermia non-invasively at depth within the body [17], and thus is ideally suited for triggered intratumoural drug release.

The TARDOX study explores the safety and feasibility of using an extracorporeal ultrasound-guided FUS device at sub-ablative powers to induce highly localized hyperthermia in liver tumours to trigger the release and enhance the delivery of systemically circulating ThermoDox®.

## Aims

This proof of concept study aims to determine whether triggered release and enhanced delivery of doxorubicin from ThermoDox® (‘drug’) using mild hyperthermia generated non-invasively by FUS is clinically feasible, safe and efficacious, for a given systemic dose.

## Methods/study design

This is a Phase I prospective non-randomised safety cohort study with all patients recruited from a single UK site (Oxford). The study has an open label design with all participants receiving systemic ThermoDox® and ultrasound-guided FUS targeted at a single target liver tumour using the Model JC200 Focused Ultrasound Tumour Therapeutic System (Haifu Medical, JC200), which is clinically approved (CE-marked) for tumour therapy in Europe and China.

The study is split into two parts. Part 1 is designed to identify optimal FUS exposure parameters for a range of tumour locations within the liver, using real-time thermometry data from an implanted thermometry device (a thermistor or thermocouple). Patients in Part 1 receive a single cycle of ThermoDox® intravenously, at a dose of of 50 mg/m^2^. After a minimum of five patients have received the Part 1 intervention with real-time thermometry, data will be reviewed by the Trial Management Group (TMG) to confirm readiness to proceed without real-time thermometry in Part 2 of the study. Subject to TMG approval, Part 2, which does not require implantation of a thermometry device, and instead uses predictions from Part 1 data to set the FUS parameters, will be opened to run in parallel to Part 1. Targeted drug delivery in Part 2 thus proceeds completely non-invasively, and this part of the study is designed to more closely reflect how the therapy might be implemented in routine clinical practice.

All participants from both Parts of the study will be included in the endpoint analysis. The primary endpoint relates to evidencing enhanced delivery of doxorubicin from ThermoDox® at the target tumour site, by comparing intratumoural concentrations of the drug before and after FUS exposure. The doxorubicin concentration will be directly determined from tissue biopsies of the target tumour, using a Good Laboratory Practice-validated high performance liquid chromatography (HPLC) assay, based on previously published methods [18, 19].

### Study population

Adult patients with incurable primary or secondary liver tumour(s) are eligible for the study.

### Inclusion criteria

- Pathologically confirmed advanced solid tumour with liver metastasis or primary liver tumour (hepatocellular carcinoma or cholangiocarcinoma). At least one liver tumour must be over 1 cm in diameter, should be visible using conventional ultrasound imaging onboard the Model JC200 Focused Ultrasound Tumour Therapeutic System and amenable to ultrasound-guided biopsy.
- Progressed or remained stable on conventional chemotherapy.
- Male or female, of age ≥ 18 years.
- Life expectancy of ≥3 months.
- Left Ventricular Ejection Fraction (LVEF) ≥ 50% on echocardiogram.
- Have not received radiotherapy to the target area within the preceding 12 months.
- World Health Organisation (WHO) performance status of ≤1.
- Able and willing to give written informed consent, indicating that they are aware of the investigational nature of this study and potential risks, and able to comply with the protocol for the duration of the study, including scheduled follow-up visits and examinations.

### Exclusion criteria

- Have surgery or other procedure requiring general anaesthesia planned to be undertaken during the period of the study.
- Serious illness including, but not limited to, congestive heart failure (NYHA class III or IV functional classification); life threatening cardiac arrhythmia; or myocardial infarction or cerebral vascular accident within the last 6 months.
- On-going significant infection (chest, urine, blood, intra-abdominal).
- Uncontrolled diabetes.
- Have received a lifetime dose of doxorubicin over 450 mg/m^2^ or a lifetime dose of epirubicin over 900 mg/m^2^, or any dose of both.
- Pregnant or breast-feeding women. In women of childbearing potential, a negative pregnancy test (serum) is required within 30 days prior to study intervention.
- Female participants of child bearing potential and male participants whose partner is of child bearing potential who are not willing to practice an acceptable form of contraception (i.e. oral contraceptive, diaphragm, cervical cap, condom, surgical sterility) during the study and for 6 months thereafter. Women whose partner has or men who have undergone a vasectomy must use a second form of birth control.
- Known allergic reactions to any of the drugs or liposomal components or intravenous imaging agents to be used in this study.
- Portal or hepatic vein tumour invasion/thrombosis.
- Any inadequate haematological or biochemical indices, as shown in Table 1.
- Have contraindications to receiving doxorubicin including prior sensitivity (rash, dyspnoea, wheezing, urticarial or other symptoms) attributed to anthracyclines or other liposomal drugs.
- Use of chemotherapy or of an investigational drug within 30 days or 5 half-lives, whichever is longer, preceding the intervention.
- Child-Pugh Class C liver disease, or Class A-B with encephalopathy and/or refractory ascites.
- HIV positive.
- Haemochromatosis.
- Contrast-induced nephropathy.
- Suspected liver haemangioma or other vascular tumour, tense ascites.
- Other medical or psychiatric conditions or laboratory abnormalities that the investigator considers would make the patient a poor trial candidate.

**Table 1:** Inadequate Biological and Haematological Indices

Laboratory Test	Exclusion Criteria
International Normalised Ratio	> 1.5 times the institution’s upper normal limit (unless anti-coagulated)
Absolute neutrophil count	<1500/mm^3^ (or <1.5 (10^9^/L))
Platelet count	< 60,000 /mm^3^ (or <60 (10^9^/L))
Haemoglobin	< 9.0 g/dL
Serum creatinine Calculated creatinine clearance (CrCl)	≥ 2.5 mg/dL (or ≥221 μmol/L) ≤ 25.0 mL/min
Serum bilirubin	> 3.0 mg/dL (or >51 μmol/L)
Serum albumin	< 2.8 g/dL (or <28g/L)

### Screening

**Fig. 1:**
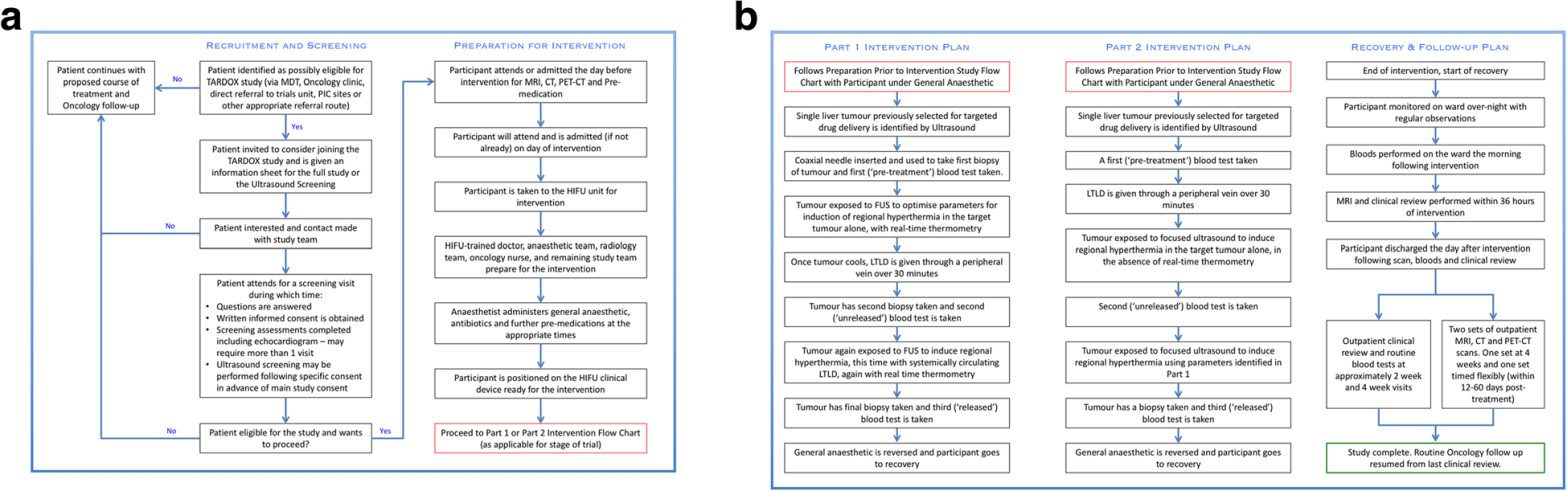
Study recruitment, screening, intervention, recovery and follow-up flow diagrams. (**a**) Flow diagrams for recruitment, screening and preparation for intervention. (**b**) Flow diagrams contrasting Part 1 and Part 2 interventions, and for recovery and follow-up

Evaluation of previous cross-sectional imaging is required to ensure availability of potential tumour targets before proceeding to screening. Screening consists of an ultrasound examination, routine blood tests, an echocardiogram and a pre-operative assessment to ensure suitability for general anaesthetic (Fig. 1a). Tumours in any liver segment will be considered for targeting, and suitability of potential target lesions will be based on ultrasound examination.

### Intervention

Baseline imaging is performed in the week prior to intervention and consists of perfusion CT, fluorodeoxyglucose (^18^F-FDG) positron emission tomography / CT (PET-CT) and dynamic contrast-enhanced MRI (DCE-MRI).

For each participant, a single liver tumour, or partial tumour volume, is targeted for drug delivery whilst under general anaesthetic (Fig. 1b). As soon as possible and for no more than 2 h after the completion of ThermoDox® infusion, the focus of the FUS device is moved through the target tumour volume in an attempt to raise the bulk tumour temperature above the thermal release threshold.

FUS-mediated hyperthermia is delivered under ultrasound guidance using clinically approved treatment modes of the therapeutic device, namely either linear (moving beam) or dot (shot-by-shot) mode, using the on-board plane-by-plane treatment planning tool to encompass all or part of the target tumour volume that can be spanned in a 30-min period. Hyperthermia is delivered with a clinically approved 0.95 MHz transducer, with a focal length of 145 mm and a diameter of 200 mm, resulting in a focus with a transverse 3-dB beam-width of 1.2 mm and an axial 3-dB length of 9.5 mm, using up to the full range of acoustic powers (32-400 W) and duty cycles (10–100%) available on the device to achieve at least 30 min of cumulative hyperthermia in the range of 40–42 °C in the target region. The specific treatment parameters will naturally vary depending on tumour anatomy.

The ability to achieve targeted release of doxorubicin from ThermoDox® at the tumour site is determined by direct analysis of tumour biopsies taken during intervention, using a GLP HPLC assay. Plasma samples obtained during the intervention are used to evaluate doxorubicin pharmacokinetics.

For Part 1 of the study, to achieve real-time thermometry of the target tumour and obtain biopsies at the same tumour site, a co-axial needle is first inserted in the target tumour under the guidance of a separate, hand-held diagnostic ultrasound system (typically Siemens S3000). This co-axial needle can be instrumented with either a thermometry probe or a biopsy needle, thus eliminating the need for repeated skin punctures and minimising patient risk.

**Fig. 2:**
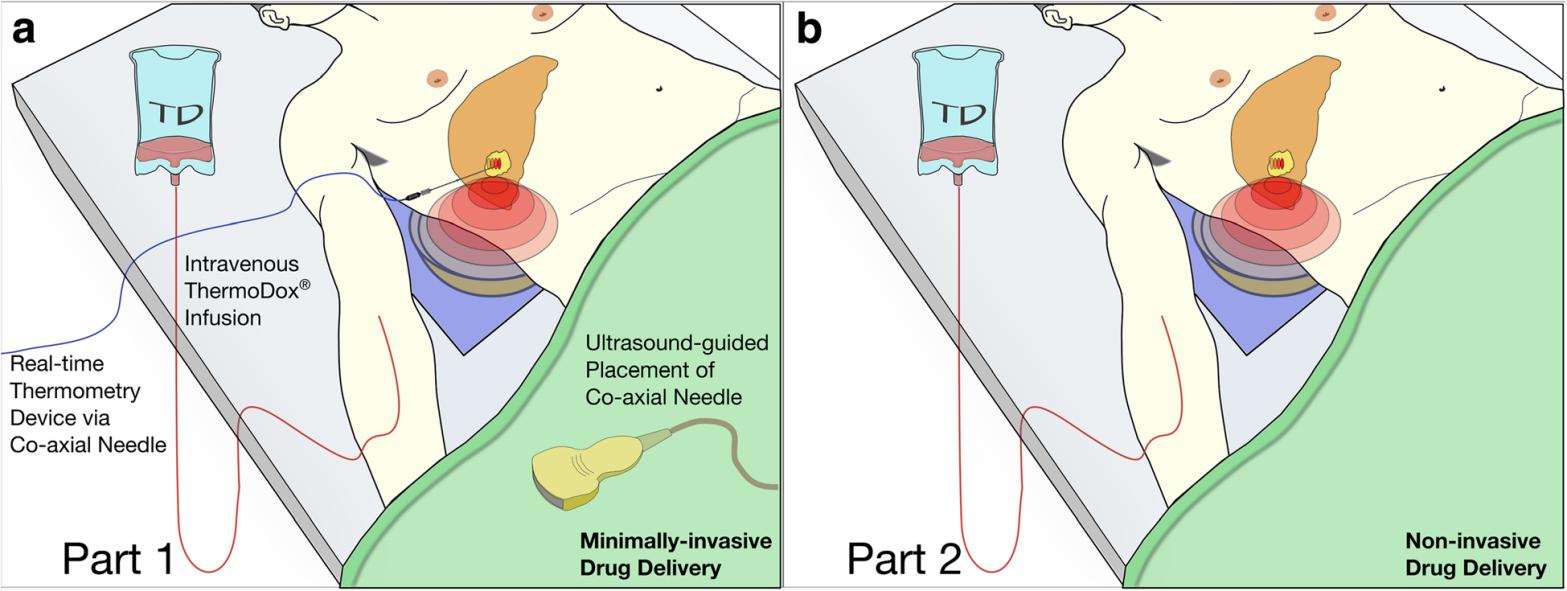
Schematics of the Part 1 (**a**) and Part 2 (**b**) study interventions highlighting the key differences between both parts of the study. The patient lies over the degassed water bath of the FUS device (JC200), which contains an ultrasound-guided therapeutic transducer, such that the focus of the transducer is aligned with the target tumour through the relevant intercostal space. (**a**) Part 1 of the study involves insertion of a co-axial needle into the target liver tumour under ultrasound guidance. This needle is used to take core biopsies of the tumour before and immediately after ThermoDox® infusion, and finally after FUS-mediated delivery, which are used in evaluation of the primary endpoint. In addition, the co-axial needle is used to pass a clinically approved thermometry device during FUS exposure, for real-time thermometry. (**b**) Part 2 does not require a thermistor, and FUS is applied to the target tumour following ThermoDox® infusion. Targeted drug delivery is thus performed completely non-invasively. Two serial core biopsies of the target tumour are taken following the FUS exposure, and tissue is used in evaluation of the primary endpoint. Part 2 affords more flexibility in patient positioning, and the supine position shown is illustrative only

With the patient under general anaesthesia, the target liver tumour is first localised using the ultrasound guidance system that is integral to the FUS device, through an intercostal space or subcostally. In order to minimise respiratory movement of the target tumour during the intervention, appropriate anaesthetic techniques, such as high-frequency jet ventilation [20], are employed. In Part 1, FUS parameters are optimised dynamically prior to drug infusion under real-time thermometry (Fig. 2a). In Part 2, in the absence of thermometry, this optimisation step is not performed (Fig. 2b) and computational models are used to predict FUS parameters (power, duty cycle, scanning speed, unit spacing) needed to achieve mild hyperthermia in the range 40–42 °C by accounting for differences in propagation path length and overlying tissue structures.

**Fig. 3:**
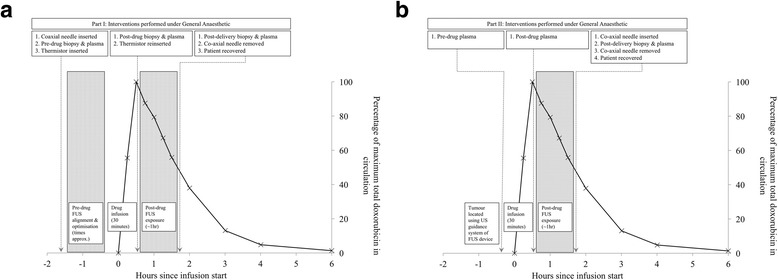
Timeline of the Part 1 (**a**) and Part 2 (**b**) interventions. Timings are appropriate and are for illustrative purposes. The pharmacokinetic curve was obtained from previous published data in patients given ThermoDox® prior to radio-frequency ablation [24]. Note that in Part 2, drug delivery is performed non-invasively and the post-delivery co-axial needle and biopsy are required purely to evaluate the primary endpoint

ThermoDox® preparation and infusion proceeds in line with the pharmacy manual provided by the manufacturer, and involves a single intravenous dose of 50 mg/m^2^ in 250 mL of normal saline or 5% dextrose over a 30-min infusion. In Part 1, biopsies of the target tumour are taken before and immediately after the drug infusion, and again following FUS-mediated delivery (Fig. 3a). When not performing a biopsy, the biopsy needle is replaced with a clinically approved implantable thermometry device inserted via the same co-axial needle (Fig. 2a). This could be either a CE-marked thermistor (Angiodynamics, Microsulis Acculis MTA Accu5i Temperature Probes, Ref: 900–314) or a CE-marked thermocouple (Medtronic, Cool-tip™ RF Ablation Remote Temperature Probe E-Series, 20 cm, Ref: RTP20). In Part 2, FUS-mediated delivery is performed non-invasively and only post-FUS delivery biopsies are taken (Fig. 3b). In both Part I and Part II, blood samples are collected immediately before the start of ThermoDox® infusion, immediately after completion of ThermoDox® infusion, and immediately after completion of FUS exposure (Fig. 3a, b).

Within the first 36 h post intervention, a DCE-MRI scan is performed for comparison with baseline. In addition to this initial imaging, follow-up imaging is performed in the first 60 days following intervention to assess potential response in the target tumour volume. Participants are subsequently followed up with a maximum of two scans of each of the following imaging modalities: CT, PET-CT and DCE-MRI. If available, other non-FUS exposed tumour(s) in the liver, which receive ThermoDox® alone, are selected as controls against which radiological response in the FUS-targeted tumour (target tumour) are compared.

The intervention, recovery and follow-up steps for both parts of the study are summarised in Fig. 1b.

### Outcome measures

The primary outcome measure for the study is the concentration of intratumoural doxorubicin at the targeted tumour site, following FUS-mediated hyperthermia. In Part 1 the comparator biopsy is taken post-drug infusion but before FUS exposure. In Part 2 the average concentration of all HPLC-evaluable Part 1 comparator biopsies is used to assess the fold-increase. To be evaluable for the primary endpoint, the tumour must have received attempted hyperthermia following drug administration. In addition, for intratumoural biopsy samples to be suitable for HPLC analysis, the tissue must be of sufficient mass, and the HPLC analytical technique must have produced validated quantifiable data.

Secondary endpoints relate to feasibility of inducing controlled FUS-mediated targeted hyperthermia in the target tumour non-invasively in Part 1 and, in addition, adverse event monitoring for 30 days in both parts. Radiological response is an exploratory (tertiary) endpoint, reflecting that only a single cycle of chemotherapy is being administered and that enhanced delivery, rather than response, is the primary aim of this early study.

### Sample size

The TARDOX study is intended to recruit up to 28 evaluable participants. No formal sample size calculation has been performed. Sample sizes have been designed to reflect the fact that this is a Phase I proof-of-concept study, and thus only small patient numbers are required. The sample size is designed to be large enough to demonstrate statistically meaningful enhanced drug delivery but small enough to ensure that participants are not unnecessarily recruited. The sample size is also in line with the available resources for this study (funding).

### Analysis plan

In Part 1, feasibility of targeted hyperthermia in the target liver tumour is assessed using an implanted thermometry device for real-time thermometry assessment. For Part 1 treatments, the cumulative equivalent in minutes (CEM43) thermal dose threshold for cell death [21] is calculated. The study aims to keep the CEM43 below 60 min, well under the ablative threshold, for each Part 1 treatment.

In addition, to explore if  any potential delayed radiological response can be attributed to a chemo-ablative process rather than direct tumour ablation secondary to high intensity FUS, a post-intervention DCE-MRI is performed with 36 h of treatment. 

The primary objective is analysed using a quantitative outcome measure, as per the primary endpoint; the concentration of intratumoural doxorubicin obtained from tumour biopsy samples.

All participants from both Parts of the study who received the intervention will be included in the primary endpoint analysis, thus evaluated on an intention to treat basis. To satisfy the primary endpoint, demonstration of a two-fold increase over the comparator, or an absolute value exceeding 10 μg/g, of the concentration of intratumoural doxorubicin at the treated tumour site following attempted FUS-induced hyperthermia, is required in at least 50% of all evaluable participants. To be included in this analysis, intra-tumoural drug concentrations from biopsy samples must have been successfully analyzed by HPLC.

Preclinical studies in a variety of human xenograft tumour types for long-circulating liposomes with a half-life of more than 10 h have demonstrated that, in the absence of ultrasound, the passive intratumoral accumulation of systemically administered liposomal doxorubicin increases by no more than 100% in the first 2 h following intravenous administration, even in the leakiest tumour type investigated [22]. In the context of ThermoDox®, a thermosensitive liposome with a much shorter half-life of around 2 h in humans [23], the criterion of a twofold increase in the intratumoural doxorubicin concentration in that same tumour following ultrasound exposure is therefore designed to identify an enhancement that is clearly attributable to FUS exposure rather than the highest possible passive accumulation over the duration of the ultrasound intervention.

Safety assessments are performed for 30 days post-intervention and consist of clinical, haematological and biochemical review. Adverse events are assessed for expectedness and causality to the drug and also to FUS, and classified according to the Common Toxicity Criteria Adverse Events (NCI CTCAE, version 4.03).

## Discussion

If this early phase study can demonstrate that ultrasound-mediated hyperthermia can safely and effectively enhance the delivery and penetration of chemotherapy agents, it could enable application of the technique to enhance therapeutic outcomes across a broad range of drug classes to treat primary and metastatic solid tumours.

## Abbreviations

CEM43: Cumulative Equivalent Minutes at 43°C
CTCAE: Common Toxicity Criteria for Adverse Events
FUS: Focused Ultrasound
GLP: Good Laboratory Practice
HIFU: High Intensity Focused Ultrasound
HPLC: High Performance Liquid Chromatography
LTSD: Lyso-Thermosensitive Liposomal Doxorubicin
